# Needs for International Benchmarking of Road Safety Management Based on Mobility Exposure Measures and Risk Patterns

**DOI:** 10.3390/ijerph182312851

**Published:** 2021-12-06

**Authors:** Guadalupe González-Sánchez, María Isabel Olmo-Sánchez, Elvira Maeso-González, Mario Gutiérrez-Bedmar, Antonio García-Rodríguez

**Affiliations:** 1 Research Group “Work and Transportation Management”, School of Industrial Engineering, University of Malaga, C/Dr. Ortiz Ramos s/n, 29071 Malaga, Spain; ggonzalez@uma.es (G.G.-S.); maribelolmo@uma.es (M.I.O.-S.); 2 Department of Public Health and Psychiatry, Instituto de Investigación Biomédica de Málaga—IBIMA, University of Malaga, 29071 Malaga, Spain; antoniogr@uma.es; 3 CIBERCV Cardiovascular Diseases, Carlos III Health Institute, 28029 Madrid, Spain

**Keywords:** road traffic injury, exposure measure, mode of transport, gender, injury severity

## Abstract

Each year, 1.35 million people worldwide die due to Road Traffic Injuries (RTI), highlighting the need for further research. The risk of RTI is usually estimated as the number of casualties divided by the level of exposure in a population. Identifying the most appropriate exposure measures is one of the most important current challenges in this field. This paper presents an analysis of exposure measures used in empirical studies on road accidents. The results show a large variability in the exposure measures used, ranging from more general measures (such as population figures or vehicle fleet) to more specific measures related to mobility (such as number of trips, distances or travel time). A comparison of the risk patterns found shows that there is a partial consensus on the profiles with the highest risk of road traffic injuries. In conclusion, there is a need for the international standardization of criteria and data to be recorded, at least injury severity and measures of exposure to mobility, as the travel time disaggregated by socio-demographic variables and mode of transport. Such data would provide higher-quality results on risk profiles and facilitate the implementation of more effective, knowledge-based road safety policies.

## 1. Introduction

Each year approximately 1.35 million people worldwide die as a result of road traffic accidents, and between 20 and 50 million suffer non-fatal injuries [1]. Although road safety strategies that were implemented in recent years are saving lives, the pace of progress is too slow. Road safety policies highlight the essential need to address road traffic injuries as a public health priority. In this regard, the United Nations General Assembly proclaimed the new Decade of Action for Road Safety from 2021–2030, with the ambitious target of halving the number of road traffic deaths and injuries by 2030 [2].

In this context, it is appropriate to deepen the analysis of Road Traffic Injury (RTI) risks in order to ensure that the most appropriate measures are taken to improve road safety. RTI is a fatal or non-fatal injury incurred as a result of a collision on a public road involving at least one moving vehicle [3]. The risk of RTI is estimated as the rate of the number of accidents or victims divided by the level of exposure of a population over a period of time. Depending on the particular objective of the analysis and the context, different rates can be used, as well as exposure data, according to their availability and quality. The need to include a reliable measure of exposure is increasingly important for road accident research.

Exposure measures can be expressed in different units (vehicle fleet, number of drivers, number of trips, distances travelled, travel times, etc.). In particular, those related to mobility are more appropriated [4]. The mobility patterns of a given population strongly influence their risk of RTI [5]. The literature suggests that there are inequalities in the risk of traffic injuries and their severity by gender, age and mode of transport [6,7]. For example, some authors refer to the necessary implementation of specific safety measures for drivers, taking into account age and gender, given the importance of these two factors in traffic accidents [8]. Other authors point out that road safety interventions should be sensitive to the sociocultural context of the behavior of specific groups of road users [9]. In this sense, vehicle speed is an important risk factor. In fact, there is evidence that large differences in speed between vehicles are related to a higher accident rate, so that vehicles traveling faster than the rest of the traffic around them have a higher risk of accidents [10]. There is evidence of a strong relationship between social class and the likelihood of road traffic injuries [11]. Therefore, it is essential to include basic demographic data such as gender and age, as well as data related to mode of transport, so that preventive measures are tailored to different groups and subgroups of road users.

Finally, the risk of road traffic injuries also depends on where people live. Many results are available on road traffic injury prevention in developed countries. However, road safety research in developing countries is scarce, especially in Africa, where the burden of road traffic injuries is high. In addition, there is an underestimation of road traffic casualties on this continent [12,13].

In this work, we carry out a comparative analysis of the different exposure measures used on empirical studies of road traffic accidents and the main findings of these measures regarding RTI risk patterns, with the aim of shedding light on the requirements for achieving better results in road traffic crash risk analysis, leading to the implementation of successful policies.

### Exposure Measures to the Risk of Road Traffic Injuries

The concept of exposure to traffic accident risk can be defined as being in a situation involving a certain risk of traffic accident [14]. In order to measure this, exposure data are used to help determine accident rates that indicate the relative degree of risk in various traffic situations.

Since 1968, there have been studies that addressed the possible causes of traffic accidents risk patterns variation, such as population, level of motorization, etc., thus introducing the importance of exposure measures in the analysis of traffic accidents [15].

Different risk estimates (rates) were used, depending on the specific purpose and context of the analysis, as well as various exposure data, depending on their availability and quality. Some exposure measures commonly used are [4]:

- In relation to vehicles: kilometers–vehicle, road length, fuel consumption and vehicle fleet;
- In relation to people: kilometers–person, population, driver census, number of trips and travel time;

Among the measures recommended as most interesting are those related to mobility, because they provide more accurate information, specifically: kilometers–vehicle, kilometers–person, number of trips and travel time [4].

The exposure to risk factors is influenced by demographic factors, socioeconomic characteristics, road type, mode of transport, etc. For example, there are significant differences in mobility patterns according to gender [16,17]. The most sustainable modes of transport such as public transport and walking are used more by women, while men make greater use of private vehicles (car and motorcycle) [18,19,20,21]. Age also influences the modal choice [22,23,24]. This shows the need to have exposure data disaggregated by gender and age for different road users. However, in practice this is not always possible. In general, the availability, quality and level of disaggregation of exposure data may be compromised by the limitations and particularities of the different collection methods. Consequently, traffic accident analysis involves some estimation (approximate) of exposure measures, which may be more or less accurate and representative of the real exposure of the population under study.

Among the sources of exposure data, mobility surveys stand out, which allow a high level of disaggregation to be obtained. However, when performed on a sample of the entire population, the data obtained are only an acceptable approximation of the real exposure. In addition, possible biases (sampling errors, lack of response or measurement) may occur. Finally, it is not always possible to conduct these surveys, given the clear difficulties in carrying them out (such as cost and time constraints), and they are limited by the lack of continuity of the measurements over time.

Given the difficulty in obtaining more accurate exposure data, other sources of exposure data are often used, such as traffic counting systems, vehicle fleet registration, driver’s licenses and/or license registrations, road network records and population records. Although these data refer to rudimentary exposure estimates, they are widely used to calculate RTI risk rates, mainly because they involve less complex collection methods and can more easily lead to comparable figures across countries.

In recent years, other methods of data collection using mobile technology were implemented, which facilitated the availability of a large amount of geolocated data over time [25,26], but did not provide information disaggregated by socio-demographic variables or modes of transport.

Another option for when exposure data are not available is to use alternative methodologies that attempt to estimate exposure indirectly from the information contained in the records of victims and traffic injuries. These methods, which were initially developed by Thorpe (1967), consider that a certain proportion of the drivers involved in traffic accidents constitute a representative sample of the driving population [27]. In general, they are known as induced or quasi-induced exposure methods. The number of users, distance traveled or travel time can be estimated indirectly as measures of exposure using a quasi-induced methodology [28,29,30,31]. However, it must be taken into account that in these methods there is a bias in the results when interpreting them.

Although the importance of including exposure in the analysis of road crash risk is recognized, in practice many studies fail to do so. For example, a systematic review of the literature on factors contributing to bicycle–motor vehicle crashes found that only 4 out of 10 studies included exposure measures. This lack of measures makes it difficult to meaningfully analyze and interpret the potential contributing factors in such crashes [32].

Therefore, regardless of the methodological approach or risk factors included in the studies, exposure data is necessary for a better interpretation of the risk of being involved in a traffic accident.

## 2. Materials and Methods

To investigate measures of exposure used in the analysis of road traffic injury risk, empirical studies on road traffic accidents that included measures of exposure were sought. We selected those that also contained basic demographic data such as gender or age, data related to mode of transport and/or degree of injury severity.

The period studied was from 1995 to 2020. The bibliographic search was restricted to scientific publications from any country published in Spanish or English. The search was conducted through articles included in the international databases Web of Science (WOS), Scopus and Medline. For this purpose, the keywords: road traffic injury, road traffic accident, gender, sex, age, mode of transport, injury severity, epidemiology, exposure measure, risk factor, mobility, road safety, crash rates, transportation, as well as synonyms and combinations of them, were entered in the “title/summary/keywords” fields. The keywords were adapted according to the web search tool used in each case.

A total of 514 publications were found in the databases considered by applying the aforementioned search strategy. After excluding duplicates, 445 results were obtained.

The relevance of the studies found for inclusion in our work was first determined by examining the title, abstract and keywords. In publications that contained basic demographic data (such as gender or age), in addition to reporting measures of exposure, mode of transport and/or degree of injury severity were filtered out. This resulted in a selection of 95 publications. Subsequently, after a more exhaustive reading of these articles, a consultation of their bibliography and a refined complementary search through Google Scholar for other articles relevant to our study, 32 papers were finally selected.

**Figure 1:**
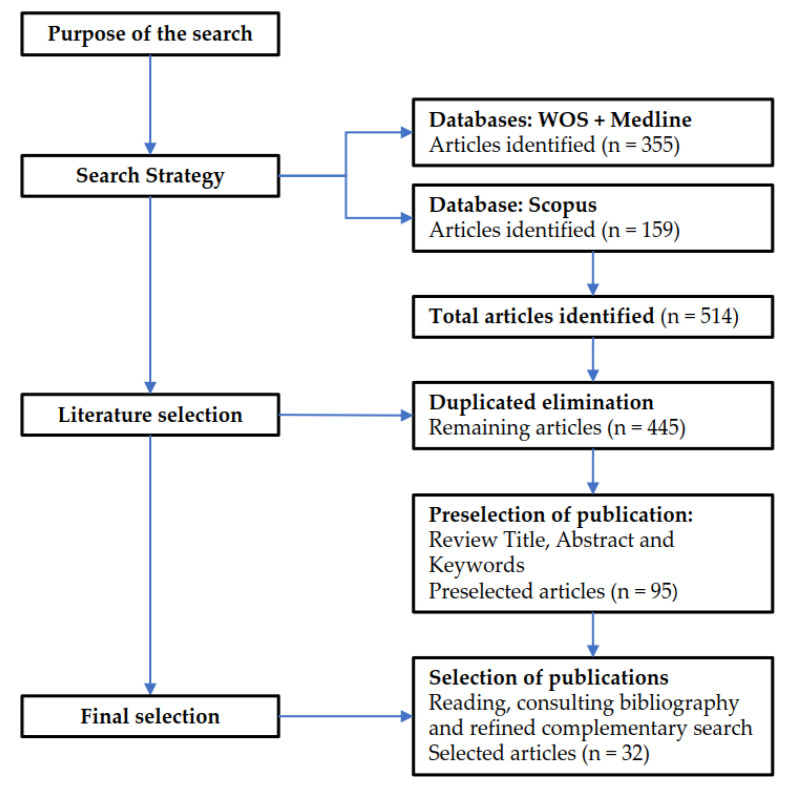
Literature review process.

Figure 1 shows the flowchart of the literature review process carried out in this study.

From these works, a comparative analysis was performed. To this end, firstly, the following were identified for each of the studies: Author and location, Exposure measure data and source, Mode of transport and Injury severity. Next, a comparative analysis of the Exposure measure data used in each study and their contribution to the achievement of quality results in the identification of traffic injury risk was performed. Afterwards, the sources used to obtain the exposure data were analyzed in order to draw conclusions on the availability, accuracy, reproducibility, harmonization, etc., of these data. Finally, the findings of these studies on Risk Patterns of Road Traffic Injury by gender, age and mode of transport were compared, distinguishing between Non-fatal and Fatal injuries, looking for coincidences and discrepancies based on the measure of exposure used.

## 3. Results

Regarding the geographical location of the works, it is found that most of them were located in Europe, although we also found a considerable number in the Americas and, to a lesser extent, in Asia and Australia. No such studies were found on the African continent. Table 1 shows the classification of the studies according to Author and location, Exposure measure data and source, Mode of transport and Injury severity.

The results of the comparative analysis of the different exposure measures used and their main findings regarding RTI risk patterns are shown below.

### 3.1. Exposure Measure Data and Source

Some authors used more general exposure measures, such as population figures, for official statistics [33,34]; the number of users of each mode of transport based on surveys [35]; the fleet of vehicles according to police databases [33]; or population figures and the driver census from national statistics [36]. Even the residence zone and the level of household income [35], or the price of gasoline and the unemployment rate were used as exposure measures [37].

Other studies used more precise measures of exposure to quantify mobility such as distances travelled from mobility survey data [38,39,40,41,42], from recorded vehicle data [43] or by estimating these distances [44]. Travel time from mobility surveys was used as a measure of exposure in a study that estimated fatality rates associated with driving and cycling [45] to study gender differences in the rates of traffic accident victims by age, mode of transport and degree of severity of the injury [6]; and to identify traffic accident risk patterns according to gender, age, mode of transport and type of road [5]. Another study on disabilities arising from traffic accidents by age, gender and type of road user also used travel time, but derived from estimates [46]. The number of trips made was also used in other studies as a measure of exposure to RTI risk, obtained from mobility survey data [47,48].

Some studies estimated and compared the risk of traffic accidents using various exposure measures, either related to mobility or other more general measures. For example, the distances travelled compared to the number of drivers [49,50], population figures and vehicle fleet [51] or population figures and number of users [52]. Others used the travel time and the number of users [53]. Another study compared the risk of RTI using travel time with others such as population census, vehicle fleet and vehicle distances travelled [54]. We can also find studies that used various exposure measures related to the mobility of people, such as distances travelled and travel times [55,56], or those that included the number of trips [57]. Another study analyzed hospital morbidity and mortality from RTI, by age and gender, comparing rates per person km and per person–hour obtained from a mobility survey, as well as rates per population [58].

Finally, given the limitation of the lack of data on mobility-related exposure measures, some authors addressed an indirect approach based on quasi-induced exposure methods to study the differences in mortality rates by the gender and age of pedestrians [28], drivers [29] or cyclists [30,31].

### 3.2. Risk Patterns of Road Traffic Injury by Gender, Age and Mode of Transport

The injury severity degrees defined in the selected studies are very different (minor, evident injury, non-fatal, disabilities resulting from traffic injury, fatal, etc.). Therefore, in order to compare their findings on the main risk profiles of traffic injuries by gender, age and mode of transport, we classified them into non-fatal and fatal injuries.

#### 3.2.1. Non-Fatal Injuries

Considering non-fatal injuries, men were found to be more at risk in cars and motorbikes [35,47] and also as cyclists, pedestrians, and public transport users [47]. It is remarkable that when introducing measures of mobility exposure, such as distance travelled or travel time, these risks vary and gender differences are attenuated, to a greater or lesser extent, depending on the variable introduced and the level of severity of the injury analyzed. Therefore, some authors found that, when considering distances, women had higher accident rates as drivers [40,41] and cyclists [55]. Studies using travel time, for minor injuries found a higher risk for women in cars, bicycles and buses [6,55], while in motorbikes and as pedestrians no gender differences were detected [6]. In contrast, for serious injuries, men continued to be at higher risk in all modes of transport [5,6], although gender differences were found to be reduced in cars, on motorbikes and as pedestrians [46]. The attenuation of gender differences is also reported in another study that compared the higher risk presented by men (without distinguishing mode of transport) when considering distances and travel times as a measure of exposure versus population numbers [58].

Continuing the analysis of non-fatal injuries by age, we found discrepancies among studies that considered distance travelled as a measure of exposure. Some identified the groups of drivers at the highest risk with the youngest [40,42], although others found the highest risk in those aged 36–50 years [49]. Other authors identified as higher risk groups, in addition to young car and motorbike users [35,46], older people in all modes of transport (car, motorbike, bicycle, pedestrian and public transport) [47]. They also found that older people were most at risk in a study of non-motorized modes (pedestrian and bicycle) [38].

Considering gender, age and mode of transport together and including distance travelled as a measure of exposure, we found that men are generally at higher risk, with gender differences being more pronounced among younger drivers [40], with this risk being reversed in favor of women for pedestrians over 65 years of age [39]. In other studies focusing on bicycle crash risk that used the number of users as a measure of exposure, the highest risk was also reported to be among men, especially younger and older men [30,31].

#### 3.2.2. Fatal Injuries

Focusing on fatal injuries, most authors agree on the higher risk for men using different measures of exposure, both general and mobility-related. This was reported both for motor vehicle drivers [33,34,36,40,41,49] and for users of all other modes of transport (pedestrian, car, motorbike, bicycle and public transport) [5,6,28,29,57]. One study reached similar conclusions, except in the case of the bus, where no significant gender differences were found [47]. A higher female risk of fatal injury was also found among pedestrians [34].

By age, young drivers show a higher fatality risk, taking as a measure of exposure the number of drivers [50], especially males when considering travel time [45], distances travelled [41] or population numbers [34]. However, other studies, taking into account distance travelled, found that older people had the highest rate of fatal crashes among drivers [40] and among pedestrians and cyclists [38]. When the number of trips, distance and time are included as measures of exposure, in addition to older people, young people presented the highest risks in all modes of transport (car, motorcycle, bicycle, pedestrian and public transport) [57].

Some authors added that gender differences increased with age across all modes of transport taking into account travel time as a measure of exposure [6] or in pedestrian fatality rates in relation to the total population [28]. A study calculating and comparing driver fatality rates using a quasi-induced exposure approach found that gender differences decreased with increasing age [29]. However, caution should be exercised in comparing these results because they are considered to be different measures of exposure.

#### 3.2.3. Other Remarks

There is also some evidence that, as the degree of severity increases, gender differences in road traffic injury risk increase, being higher for men [6].

Injury severity is positively associated with age in all modes of transport, except for motorbikes, where the opposite is true [5].

Finally, it should be noted that, in addition to gender, age and mode of transport, some studies also include other types of risk factors. For example, the age of driver’s license [36] and external environmental factors such as weather conditions, road surface conditions, and visibility conditions [49], type of road [5,36], light condition or time of day [40,41], socioeconomic level of the town or residence area [48,52], etc. We also found some research that not only focuses on identifying road traffic injuries patterns, but also analyses trends over the years [33,34,38,44].

## 4. Discussion

This work examined the risk exposure measures used in empirical studies of road traffic accidents and over their main findings on RTI risk patterns.

The literature search provided us with a small number of studies that took into account measures of exposure related to mobility and considered variables such as gender, age, mode of transport or severity of injury. This is despite the fact that the greater utility of these exposure measures is well known [4]. In particular, it was observed that the exposure measures that provide the best results are those that quantify the mobility of people rather than the mobility of vehicles, since these factors make it possible to identify the groups at the greatest risk, compare regions with different mobility patterns, and assess trends in traffic accidents as a function of changes in mobility patterns [54]. Among the exposure measures related to people’s mobility, travel time, distances travelled and number of trips stand out. However, the first two measures provide more complete and accurate information on actual exposure than the latter, which only offers data on modal split. Some authors point out that the relationship between space, time and risk is complex, and that speed plays an important role in this relationship, recommending that exposure should be measured by a combination of space travelled and travel time [59]. The results obtained in two of the selected studies comparing RTI rates, which used both measures of exposure, concluded that the time indicator provided a more accurate estimate because it was more adaptable to all types of situations and more flexible in relation to different environmental and personal risk circumstances [54,58].

Among the main reasons for the lack of such studies with measures of mobility exposure may be the difficulty of obtaining actual disaggregated exposure data for different groups and subgroups of road users. In the absence of data, some studies chose to estimate travel times [46], distances covered [44], or both [28]. To overcome this impediment, it is necessary to improve the regular and systematic recording of statistical data on people’s mobility, disaggregated by demographic, socioeconomic, different modes of transport and road environment variables. A comparative study in Europe on methodological approaches in the analysis of road safety trends noted that there is a technical limitation due to lack of data rather than lack of appropriate methods or techniques [60]. Although progress is being made in the development of common and harmonized databases on road accidents such as the International Traffic Safety Data and Analysis Group (IRTAD) [61] or the European Road Safety Observatory (ERSO) [60], information is still mainly missing from exposure data from many countries and/or not sufficiently standardized. Further research is needed on exposure measurements, as well as ways to collect and standardize measurements worldwide. This need is even more pronounced in developing and underdeveloped countries [13]. Mobility surveys traditionally collect this type of data, and, nowadays, interesting opportunities have arisen based on the potential of new technologies, the use of smartphones and MaaS (Mobility as a Service) applications, revealing very accurate information on people’s mobility patterns [62].

A comparison of the main findings on RTI risk patterns by sex, age, mode of transport and injury severity reveals a partial consensus on certain groups at particular risk of RTI. Highlighting the higher risk of fatal injuries for men, in general, in all modes of transport. The gender and age differences found in RTI risk patterns confirm the importance of standardizing these variables in the design of mobility and road safety policies, as well as in their implementation and management [21]. Furthermore, the correct identification of higher risk groups could help to develop Intelligent Transport Systems (ITS) that reduce risk exposure, and thus improve road safety [63].

Emphasizing that the comparison of findings on RTI risk patterns was complicated because, the different study populations with different modes of transport (drivers and/or passengers of cars, motorcycles, mopeds, bicycles, pedestrians, users of public transport, etc.) and the origin of the traffic accident data (surveys, official police records, hospitalized patient records, etc.), as well as the definitions of the degrees of injury severity, varied widely. A study on the needs and priorities of road safety stakeholders for evidence-based policy-making development identified the importance of establishing common definitions of serious injury and fatality, as well as crash databases linking police and hospital casualty data [64]. Whereas international comparisons of road safety based on fatalities have a long tradition and established practice, international comparisons of serious injuries are difficult to make due to different definitions of serious injuries between and within countries [65]. In the European Union, the MAIS 3+ definition of serious injury is widely used. MAIS is a globally accepted injury severity classification scale used by medical professionals ranging from 1 to 6, with levels 3 to 6 considered as serious injuries [66]. A study conducted in Sweden, the Netherlands and Finland highlighted the importance of using an indicator combining Killed and Seriously Injured (KSI) and the need to define guidelines for the collection, reporting and analysis of international data on serious injuries [65]. To contribute to the benchmarking of countries’ road safety performance, some researchers advanced the design of a composite road safety indicator based on the different components of the road safety pyramid, also highlighting the need to develop high-quality and comparable data collection procedures [67].

Finally, the influence of mobility patterns on the risk of RTIs reveals the need to consider mobility and road safety policies in an integrated way. A study on the interactions of environmental and safety measures for sustainable road transport found that a large majority of the measures examined supported both policy objectives; however, there were also measures with contradictory effects, e.g., environmental measures such as increased cycling and walking were likely to have negative effects on road safety [68]. Another study on pedestrian injury risk highlighted that pedestrian safety must mean more than not being hurt; it was also necessary to focus on the creation and maintenance of an inclusive, comfortable and welcoming environment [69]. Therefore, sustainable mobility policies that promote the use of non-motorized modes such as walking and cycling, must be accompanied by strategies designed to ensure road safety in these vulnerable modes.

As limitations of this work we can mention that, despite the fact that the studies were searched in four international databases which are highly recognized by the scientific community, there may be publications related to the topic that were not contemplated in this study. An interesting direction for future research, in line with the limitations noted above, would be to use other databases and consider including practical guides, specialized books and grey literature to obtain a more complete overview of the use of exposure measures in road crash analysis.

## 5. Conclusions

The identification of appropriate exposure measures is key in the analysis of RTI risk and constitutes one of the most important current challenges in the investigation of road accidents and their victims.

The analysis of a selection of empirical studies on road accidents with reported exposure measures shows a wide variety of indicators used. Some of these are more general (population figures, vehicle fleet, etc.) while others are related to mobility (number of trips, distances travelled or travel time). It can be pointed out that the latter are more interesting because they provide higher-quality results. Particularly, distances and time give more complete and accurate information on actual exposure, with travel time being the more versatile measure.

Although the need to include exposure measures is generally recognized in road accident research, reliable data are often unavailable.

The comparative analysis of the findings on RTI risk patterns by gender, age, mode of transport and injury severity reveals that there is a partial consensus on the highest risk profiles although it is difficult to contrast study results due to the diversity of variables, data sources, variety of definitions on injury severity degrees, population under study and, of course, the exposure measures considered.

An international framework for science-based analysis and policy development is essential. In particular, there is a need to unify criteria for classifying injury severity and to collect data that would allow the application of RTI rates based on measures of mobility exposure, at least travel time disaggregated by socio-demographic and mode of transport variables. This would allow for a better identification of higher risk groups and also for effective comparisons between geographical locations, taking into account their particular mobility patterns. Consequently, it could help in identifying countries with successful road safety programs and the best practices for road traffic injury prevention.

## Figures and Tables

**Table 1:** Empirical studies on traffic accidents with reported exposure data.

Author and Location	Exposure Measure Data and Source	Mode of Transport	Injury Severity
Al-Balbissi, 2003 Jordan (Asia)	Drivers, distance (mobility survey)	Motor vehicles drivers	Property damage, injury, fatal
Aparicio Izquierdo et al., 2017 Spain (Europe)	Population figures, drivers (Statistics National Institute, National Traffic Authority)	Car	Serious, fatal (injury severity to occupants of vehicles or pedestrians)
Babanoski et al., 2016 Republic of North Macedonia (Europe)	Population figures, number of vehicles, distance (State Statistical Office)	Not specified	Fatal
Bahadorimonfared et al., 2013 Iran (Asia)	Population figures, number of vehicles (databases of police records)	Motor vehicles	Fatal
Beck et al., 2007 USA	Person–trip (National Household Travel Survey)	Passenger vehicle, motorcycle, walking, bicycle, bus, other vehicles	Fatal, nonfatal
Berecki-Gisolf et al., 2015 Thailand (Asia)	Number of users, area of residence, household income (Surveys from Thai Cohort Study)	Car, motorcycle	Nonfatal
Buehler and Pucher, 2017 USA and Germany (Europe)	Distance (mobility surveys)	Walking, bicycle	Serious, fatal
Ferrando et al., 1998 Barcelona, Spain (Europe)	Population figures, time (estimates from Barcelona Health Questionnaire)	Car users, motorcycle users, pedestrian, other (public transport) users	Disabilities resulting from traffic injuries
González-Sánchez et al., 2018 Andalucía, Spain (Europe)	Time (Mobility Survey in Andalucía)	Pedestrian, car users, motorcycle/moped users, bicycle, public transport passengers	Total, minor, serious, fatal
Haddak, 2016 France (Europe)	Number of trips, distance, time (French National Travel Survey)	Pedestrian, Cyclists, motorized two-wheeler users, car occupants, public transport	Fatal
Li et al., 1998 USA	Drivers, distance (mobility survey)	Motor vehicles	Fatal
Licaj et al., 2011 Rhône Département, France (Europe)	Population figures, number of users, distance (mobility survey)	Car as passenger, car as driver, motorized two wheelers, public transport, bicycle	Total
Lovelace et al., 2015 West Yorkshire, UK (Europe)	Distance (estimates based on census commuting statistics)	Bicycle	Serious, fatal
Majdan et al., 2015 Austria (Europe)	Population figures (central statistical office of Statistics Austria)	Pedestrian, motor vehicle drivers, motor vehicle passengers, motorcycle driver/passenger, bicyclist, unspecified user	Fatal
Malin et al., 2020 Finland (Europe)	Distance (Finnish National Travel Survey)	Pedestrian	Serious and fatal
Martínez-Ruiz et al., 2014 Spain (Europe)	Number of users (estimates based on a quasi-induced methodology)	Bicycle	Serious or fatal, minor,
Martínez-Ruiz et al., 2015 Spain (Europe)	Number of users (estimates based on a quasi-induced methodology)	Bicycle	Fatal
Massie et al., 1995 USA	Distance (Nationwide Personal Transportation Survey)	Motor vehicles drivers	Fatal, injury, property-damage
Massie et al., 1997 USA	Distance (Nationwide Personal Transportation Survey)	Motor vehicles drivers	Fatal, injury, property-damage
Obeng, 2011 Greensboro, North Carolina, USA	Gasoline price, unemployment rate (Greensboro Department of Transportation)	Passenger car, sports utility vehicle, van, pickup	Fatality, incapacitating, evident injury, possible injury, no injury (property damage only)
Onieva-García et al., 2016 Spain (Europe)	Distance, time (estimates based on decomposition and quasi-induced exposure methods)	Pedestrian	Fatal
Paefgen et al., 2014 (Europe)	Distance (in-vehicle data recorders –IVDR- database)	Motor vehicles	Incident, incident with injuries, incident with death
Papa et al., 2014 Naples, Italy (Europe)	Distance (mobility survey)	Motor vehicles drivers	CIRS-SI = Cumulative Illness Rating Scale-Severity Index, CIRS-CI = Cumulative Illness Rating Scale-Comorbidity Index
Pirdavani et al., 2017 Flanders, Belgium (Europe)	Number of trips (mobility survey)	Car driver, car passenger, active mode user (pedestrians and cyclists)	Injury, fatal crashes
Poulos et al., 2015 New South Wales (Australia)	Distance, time (mobility survey)	Bicycle	Crash without injury, self-treated injury, medical attention injury (but not an overnight stay in hospital), hospitalized injury (requiring an overnight stay in hospital)
Poulos et al., 2017 New South Wales (Australia)	Distance, time (mobility survey)	Bicycle	Crash without injury, self-treated injury, medical attention injury (but not an overnight stay in hospital), hospitalized injury (requiring an overnight stay in hospital)
Pulido et al., 2016 Spain (Europe)	Number of users (estimates based on a quasi-induced methodology)	Car drivers	Fatal
Sá et al., 2016 São Paulo, Brazil (America)	Number of users, time (Sao Paulo Household Travel Survey)	Bicycle	Fatal, nonfatal
Santamariña-Rubio et al., 2013 Catalonia, Spain (Europe)	Population census, vehicle fleet, time, vehicles distances (National Statistics Institute, Catalonian Daily Mobility Survey, Ministry of Public Works)	Car, motorcycle/moped, bus, truck/van	Total
Santamariña-Rubio et al., 2014 Catalonia, Spain (Europe)	Time (Catalonian Daily Mobility Survey)	Pedestrian, car drivers, motorcycle/moped drivers, bicycle, bus passengers	Total, minor, serious, fatal
Scholes et al., 2018 UK (Europe)	Time (National Travel Survey)	Car drivers, cyclists	Fatal
Velázquez-Buendía et al., 2015 Community of Madrid, Spain (Europe)	Population figures, distance, time (Mobility House Survey of the Community of Madrid)	Not specified	Fatal, hospital discharges

## Data Availability

Not applicable.
